# Robot-assisted technique versus freehand technique for spine surgery: an umbrella review

**DOI:** 10.1080/07853890.2025.2523564

**Published:** 2025-07-09

**Authors:** Ting Li, Jingxin Yan, Jin Li, Yuanting Shang, Xiaoyu Tang

**Affiliations:** a Department of Orthopedics, No.1 Orthopedics Hospital of Chengdu, Chengdu, Sichuan, China; b School of Medicine, South China University of Technology, Guangzhou, China

**Keywords:** Free-hand, meta-analysis, robot-assisted, systematic review, spine surgery, umbrella review

## Abstract

**Background:**

Pedicle screw internal fixation is a significant method for maintaining spinal stability. Despite the existence of numerous systematic reviews and meta-analyses that have evaluated the efficacy and safety of robotic-assisted compared to freehand technique, a comprehensive evaluation of the evidence’s strength and quality remains to be conducted.

**Methods:**

A comprehensive search of five major databases (i.e. PubMed, Embase, Cochrane, Web of Science, and Scopus) was conducted up to May 2024. The present umbrella review was conducted in accordance with the Preferred Reporting Items for Systematic Reviews and Meta-Analysis (PRISMA) guidelines. The quality of the included meta-analysis was evaluated by AMSTAR-2 (A Measurement Tool to Assess Systematic Reviews). The quality of the evidence for each association was evaluated using the Grading of Recommendations, Assessment, Development, and Evaluations (GRADE) approach.

**Results:**

The present study incorporated a total of 22 meta-analyses, encompassing randomized clinical trials, cohort studies, and observational studies. A total of 14 associations with ‘perfect’ pedicle screw, ‘clinically acceptable’ pedicle screw, complications, facet joint violation, intraoperative radiation dose, intraoperative radiation time, and other related outcomes were evaluated in this umbrella review. Robot-assisted techniques in spine surgery have been found to be beneficial. The quality of the majority of the associations ranged from low to very low, indicating that flaws were certain in the current meta-analyses.

**Conclusion:**

This umbrella review indicated potential advantages of robotic-assisted technique for spine surgery, despite these findings with low certainty of the evidence, and cautious interpretation and application in clinical practice are needed.

## Introduction

Pedicle screws have been extensively adopted by orthopaedic and neurosurgical practitioners in spine surgery due to their proven advantages. Based on the three-column theory of the spine, using pedicles in spinal reconstruction offers a strong stability foundation [1,2]. Indications for pedicle screw fixation include spinal degenerative dystrophic diseases (affecting 80% of the population), spinal trauma (5–6% of all fractures), and spinal neoplasms (predominantly metastatic, with primary tumors demonstrating an annual incidence of 0.74–2.5 per 100,000) [2–4]. Pedicle screws are frequently utilized in the thoracic, lumbar, and cervical regions of the spine [5,6]. However, the thoracic region is characterized by the highest risk of misplacement, which can be attributed to the anatomical characteristics of Pericles [7]. Research has documented misplacement rates ranging from 5% to 41% in the lumbar spine, 3% to 55% in the thoracic spine, and 3% to 25% in the cervical spine [7–9].

In order to enhance the accuracy of pedicle screw placement, the traditional free-hand technique has been supplemented by robot-assisted navigated spine surgery [10]. Whilst some meta-analyses and systematic reviews have indicated that robot-assisted navigation improves pedicle screw accuracy, D’Souza et al. suggested that there was no significant difference when compared to conventional free-hand methods [11]. A plethora of meta-analyses of RCTs have been conducted, and these demonstrate that pedicle screw placement is more accurate when the robot-assisted technique is employed as opposed to free-hand methods. Nevertheless, there is persistent clinical heterogeneity regarding operative time, complication rates and radiation exposure across studies [12–15]. It is noteworthy that Lee et al. [16] have demonstrated that Level I evidence indicates equivalent radiation safety profiles and clinical outcomes between modalities. However, additional uncertainty persists with regard to cost-effectiveness and hospitalization metrics [17,18]. Consequently, conducting an umbrella review is imperative to evaluate the overall quality of the extant evidence on this subject. An umbrella review, in essence a review of reviews, systematically collects and synthesizes higher levels of evidence in order to provide a comprehensive evaluation of the available evidence.

## Materials and methods

### Umbrella review methods and registration

The present umbrella review was conducted in accordance with the Preferred Reporting Items for Systematic Reviews and Meta-Analyses (PRISMA: http://www.prisma-statement.org) [19] guidelines, and was registered with PROSPERO (ID: CRD42023471380).

### Search strategy

A comprehensive search was conducted across five electronic databases: PubMed, Scopus, Embase, the Cochrane Database, and Web of Science, with the search period extending up to May 2024. Moreover, the present umbrella review was exclusively concerned with English-language literature, and its primary objective was to conduct a comparative analysis of robot-assisted surgery and the free-hand technique. The search strategy is outlined in detail in Supplementary Table S1.

### Selection criteria

The eligible studies were systematic reviews and meta-analyses that evaluated the clinical outcomes of robot-assisted versus free-hand technique for spinal disease treatment.

The inclusion criteria for this study encompassed spinal diseases, including spinal deformity (scoliosis and kyphosis), revision surgery, degenerative pathologies (e.g. spondylolisthesis and spinal stenosis), spinal trauma (non-emergent cases), and neoplasms. In addition to the surgical sites, the following vertebrae were included: the cervical, thoracic, and lumbar.

The exclusion criteria were as follows: (1) other’s reviews; (2) articles lacking outcome evaluations; (3) utilization of robot-assisted PKP (percutaneous kyphoplasty) or PVP (percutaneous vertebroplasty) for the treatment of vertebral compression fractures; (4) additionally, the authors excluded studies involving robot-assisted and navigation-assisted screw placement procedures. Navigation-assisted pedicle screw fixation, while a valuable intraoperative guidance modality, was excluded from comparative analysis for methodological consistency. This decision aligns with PRISMA guidelines for umbrella reviews, which prioritize homogeneity in synthesizing evidence from pre-existing systematic reviews and meta-analyses; (5) no-English.

### Data extraction and quality assessment

In accordance with the established inclusion and exclusion criteria, two independent researchers were tasked with the review and extraction of data from a series of potential studies. The extracted data comprised study details, including author, year, participant numbers, interventions, outcomes, and so on. The outcome measures reported in the original meta-analyses were also collated.

The accuracy of pedicle screw placement was assessed using the Gertzbein–Robbins grading scale: Grade A: Screw entirely within the pedicle cortex (no breach); Grade B: Pedicle breach ≤2 mm; Grade C: Pedicle breach 2–4 mm; Grade D: Pedicle breach 4–6 mm; Grade E: Pedicle breach >6 mm. Grade A was defined as a ‘perfect screw’. If the screw was ≤ 3 mm outside the pedicle without relevant complications (Grade A + B), we categorized them as ‘clinically-acceptable’ accuracy [20].

Two independent reviewers used AMSTAR2 to assess the methodological quality of included articles. The AMSTAR 2 tool evaluates the quality of systematic reviews and meta-analyses based on 16 specific items. Below is a detailed list of the 16 items: 1. Protocol and Registration; 2. Blinding of Reviewers to Study Results; 3. Duplicate Study Selection and Data Extraction; 4. Comprehensive Literature Search; 5. Search for Grey Literature; 6. Inclusion Criteria; 7. Demonstration of Criteria Application; 8. Study Quality Assessment; 9. Risk of Bias (ROB) Assessment; 10. Publication Bias Assessment; 11. Adequate Statistical Methods; 12. Heterogeneity Assessment; 13. Assessment of Confidence in the Evidence (GRADE); 14. Incorporation of Evidence from Different Groupings; 15. Justification of Exclusions; 16. Conflict of Interest Disclosure (Yes/No/Cannot Answer). The AMSTAR2 questionnaire uses 16 measures to classify systematic reviews as high, moderate, low, or critically low quality.

### Data synthesis and analysis

Data extraction and analysis were performed using R software version 4.6. The certainty of the evidence was evaluated using GRADE (http://www.gradepro.org). The quality of the evidence was graded as ‘high’, ‘moderate’, ‘low’, or ‘very low’ based on the following characteristics: inconsistency between studies (I^2^ statistic), study design (observational, randomized clinical trials), imprecision, publication bias, and the presence of a dose–response gradient. Recommendations were also provided according to these grades.

## Results

### Search result

**Figure 1.**
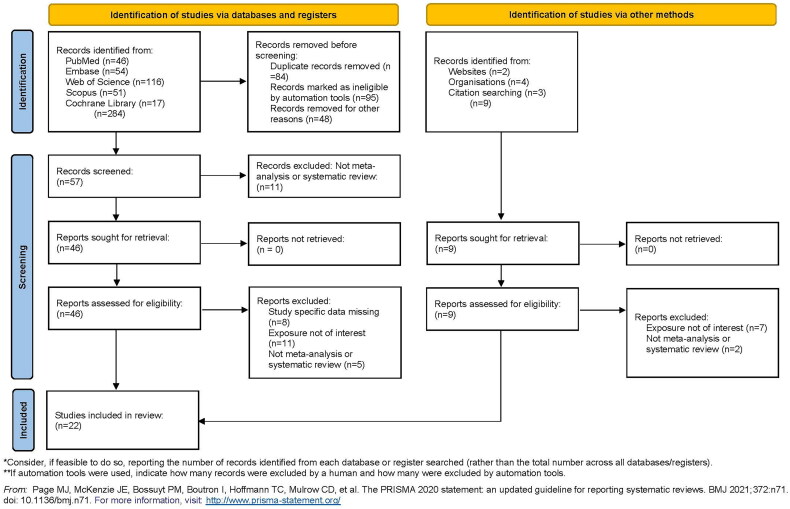
PRISMA 2020 Flow diagram for new systematic reviews which included searches of databases, registers, and other sources.

As illustrated by Figure 1, a PRISMA flowchart was employed to depict the process of literature searching. The preliminary search yielded a total of 293 articles, of which 84 were deemed to be duplicates and thus excluded from further consideration. A total of 18 non-meta-analysis and systematic review studies were excluded, and 143 articles were excluded for other reasons. Following a comprehensive screening and assessment process against predefined inclusion criteria, 22 studies [21–42] were identified as eligible and incorporated into the umbrella review. Table 1 provides a comprehensive summary of the characteristics of the included studies, including details such as study objectives, the number of studies per category, and the most significant results obtained. It is noteworthy that seven of these studies exclusively comprised RCTs, while the remaining studies included a combination of observational studies and RCTs.

**Table 1.** Basic characteristics of the included literature.

Name	Comparison of groups	Total number of studies (participants included in each meta-analysis)	Results
Fan [21]	Robotic-assisted technique vs. free-hand with fluoroscopy-guided technique	5 RCTs, 4 retrospective cohort studies, and 1 prospective cohort study (*n* = 609 patients) (included: Thoracic, Lumbar, Lumbosacral)	1. ‘Perfect’ pedicle screw, 2. ‘Clinically acceptable’ pedicle screw
Fatima [22]	Robotic-assisted technique vs. free-hand technique	7 RCTs, 10 retrospective comparative studies, and 2 prospective cohort studies (*n* = 1525 patients) (included: Thoracic, Lumbar, Lumbosacral)	1. ‘Perfect’ pedicle screw, 2. ‘Clinically acceptable’ pedicle screw, 3. Complications, 4.FJV. 5. IRD and IRT, 6. OT
Fu [23]	Robotic-assisted technique vs. free-hand technique	15 RCTs (NA) (included: NA)	1. ‘Perfect’ pedicle screw, 2. LOS, 3. IBL, 4. FJV. 5. IRD, 6. OT, 7. VAS, 8. ODI
Gao [24]	Robotic-assisted technique vs. free-hand technique	6 RCTs (*n* = 306 patients) (included: Lumbar)	1. ‘Perfect’ pedicle screw, 2. ‘Clinically acceptable’ pedicle screw, 3. FJV, 4. IRD and IRT, 5. OT
Himstead [25]	Robotic-assisted technique vs. free-hand technique (fluoroscopy-guided, O-arm Navigation, CT-navigation, navigational template)	11 RCTs, 20 retrospective cohort studies, and 4 prospective cohort studies (*n* = 3130 patients) (included: NA)	1. ‘Clinically acceptable’ pedicle screw, 2. OT, 3. LOS, 4. IBL
Li [26]	Robotic-assisted technique vs. free-hand with fluoroscopy-guided technique	13 RCTs (*n* = NA) (included: Lumbar)	1. ‘Perfect’ pedicle screw, 2. Complications, 3. FJV, 4. Revision rate, 5. RT (per screw)
Li [27]	Robotic-assisted technique vs. free-hand technique	9 RCTs (*n* = 696 patients) (included: Thoracolumbar, Lumbar)	1. ‘Perfect’ pedicle screw, 2. Grade B pedicle screw, 3. Grade C + D+E pedicle screw, 4. OT, 5. LOS, 6. IRD and IRT, 7. VAS, 8. ODI
Li [28]	Robotic-assisted technique vs. free-hand technique	5 RCTs, 15 retrospective cohort studies, and 3 prospective cohort studies (*n* = 2520 patients) (included: NA)	1.IRD and IRT, 2. OT, 3. LOS, 4. Complications, 5. Revision rate
Li [29]	Robotic-assisted technique vs. free-hand with/without fluoroscopy-guided technique	10 RCTs (*n* = 713 patients) (included: Thoracolumbar, Lumbar, Sacral)	1. ‘Perfect’ pedicle screw, 2. ‘Clinically acceptable’ pedicle screw, 3. IRD and IRT, 4. OT, 5. FJV, 6. IBL, 7. VAS, 8. LOS, 9. ODI
Li [30]	Robotic-assisted technique vs. free-hand technique	4 RCTs, and 4 retrospective cohort studies (*n* = 508 patients) (included: NA)	1. VAS, 2. ODI, 3. OT, 4. IBL, 5. LOS
Liu [31]	Robotic-assisted technique vs. free-hand technique	3 RCTs, 1 retrospective cohort study, and 1 prospective cohort study (*n* = 257 patients) (included: Lumbar, Lumbosacral)	1. ‘Perfect’ pedicle screw, 2. Grade B pedicle screw
Luengo–Matos [32]	Robotic-assisted technique vs. free-hand technique	11 RCTs (*n* = 817 patients) (included: Lumbar, Lumbosacral)	1. ‘Perfect’ pedicle screw, 2. ‘Clinically acceptable’ pedicle screw, 3. FJV, 4. IRD and IRT.5, OT, 6. LOS,7 IBL
Naik [33]	Robotic-assisted technique vs. free-hand technique	17 studies (*n* = NA) (included: NA)	1. ‘Perfect’ pedicle screw, 2. Complications, 3. OT, 4. IBL, 5. LOS, 6. VAS, 7. ODI
Peng [34]	Robotic-assisted technique vs. free-hand technique	7 RCTs (*n* = 540 patients) (included: Thoracolumbar, Lumbar)	1. ‘Perfect’ pedicle screw, 2. ‘Clinically acceptable’ pedicle screw, 3. Grade C + D+E pedicle screw, 4. OT, 5. IRT
Siccoli [35]	Robotic-assisted technique vs. free-hand technique	4 RCTs, 4 retrospective cohort studies, 1 prospective cohort study (*n* = NA) (included: Thoracic, Lumbar, Lumbosacral)	1. IRD, 2. OT, 3. LOS, 4. Complications, 5.IBL
Staartjes [36]	Robotic-assisted technique vs. free-hand with technique	3 RCTs, 4 retrospective cohort studies, 2 prospective cohort studies (*n* = NA)	1. revision rate.
Tarawneh [37]	Robotic-assisted technique vs. free-hand technique	7 RCTs (*n* = 290 patients) (Included: NA)	1. ‘Perfect’ pedicle screw, 2. ‘Clinically acceptable’ pedicle screw, 3. Grade C + D+E pedicle screw, 4. OT, 5. IRT and IRD, 6. LOS, 7. VAS, 8. Revision rate
Wei [38]	Robotic-assisted technique vs. free-hand technique	26 studies (*n* = NA) (Included: NA)	1. ‘Perfect’ pedicle screw, 2. ‘Clinically acceptable’ pedicle screw, 3. Complications, 4. FJV, 5. IRT and IRD
Yu [39]	Robotic-assisted technique vs. free-hand technique	3 RCTs, and 6 retrospective comparative studies (*n* = 750 patients) (Included: NA)	1. ‘Clinically acceptable’ pedicle screw, 2. Complications, 3. OT, 4. IRT
Zhou [40]	Robotic-assisted technique vs. free-hand technique	3 RCTs, 2 prospective cohort studies, and 1 retrospective cohort study (*n* = 783 patients) (Included: NA)	1. FJV, 2. Grade C + D+E pedicle screw, 3. Revision rate, 4. IRD, 5. OT
Zhou [41]	Robotic-assisted technique vs. free-hand with computer-assisted navigation technique	1 RCT, and 5 retrospective cohort study (*n* = 529 patients) (Included: Thoracolumbar)	1. ‘Perfect’ pedicle screw, 2. ‘Clinically acceptable’ pedicle screw, 3. OT, 4. IBL, 5. Complications, 6. Revision rate
Zhou [42]	Robotic-assisted technique vs. free-hand technique	1 RCT, 3 comparative cohort studies, and 3 case series consisting (*n* = 160 patients) (included: Cervical)	1. ‘Perfect’ pedicle screw, 2. ‘Clinically acceptable’ pedicle screw

FJV: facet joint violation; IRD: intraoperative radiation dose; IRT: intraoperative radiation time; OT: operative time; LOS: length of stay; IBL: intraoperative blood loss; VAS: visual analogue scale; ODI: Oswestry disability index.

### Outcome of quality assessment

As illustrated in Table 2, the AMSTAR2 item evaluations were employed to assess the included meta-analyses. Of the 22 studies that were included in the analysis, three were rated as moderate in quality, ten were rated as low, and nine were rated as critically low. Specifically, the majority of the included meta-analyses (11 out of 12) utilized appropriate methods for statistical analysis (Q11). However, it is evident that none of the review authors provided a list of excluded studies or adequately justified the reasons for their exclusion. The odds ratios (OR), relative risks (RR), mean differences (MD), and standardized mean differences (SMD) for the most significant outcomes are documented in Supplementary Tables S2. The GRADE framework categorized the certainty of the evidence as low to very low for the majority of outcomes. The following specific factors contributed to this downgrading: The limitations of the original RCTs can be categorized as follows: methodological limitations (1) substantial heterogeneity among studies (2) inconsistency (3) imprecision due to either wide confidence intervals or small sample sizes (4) and potential publication bias (Supplementary Tables S2).

**Table 2.** The results of the methodological quality assessment of the meta-analysis: item-by-item quality rating using the AMSTAR-2.

First author et al.	Year	Q1	Q2	Q3	Q4	Q5	Q6	Q7	Q8	Q9	Q10	Q11	Q12	Q13	Q14	Q15	Q16	Quality assessment
Fan, Y	2018	N	Y	Y	PY	N	Y	Y	Y	Y	N	Y	Y	Y	Y	Y	N	Low
Fatima, N	2021	Y	Y	Y	PY	N	Y	Y	Y	Y	N	Y	Y	Y	Y	Y	N	Low
Fu, WG	2021	N	Y	Y	PY	N	N	Y	N	Y	N	Y	Y	Y	Y	N	Y	Critically low
Gao, ST	2018	Y	PY	Y	Y	Y	Y	Y	Y	Y	N	Y	Y	Y	Y	Y	Y	Moderate
Himstead, AS	2022	Y	Y	Y	PY	Y	Y	Y	Y	Y	N	Y	N	N	Y	N	Y	Critically low
Li, CT	2021	N	N	N	PY	N	N	Y	Y	Y	N	Y	Y	N	Y	N	N	Critically low
Li, HM	2020	Y	Y	Y	Y	Y	Y	Y	Y	Y	N	Y	Y	Y	Y	N	N	Low
Li, JY	2020	Y	PY	N	PY	Y	N	Y	Y	Y	N	Y	N	Y	Y	N	N	Low
Li, W	2020	N	N	Y	PY	N	Y	Y	Y	Y	N	Y	N	N	N	N	N	Critically low
Li, YY	2023	N	Y	Y	PY	Y	N	Y	Y	Y	N	Y	Y	Y	Y	Y	Y	Low
Liu, H	2016	Y	PY	Y	PY	Y	Y	Y	Y	Y	N	Y	Y	Y	Y	Y	Y	Critically low
Luengo–Matos, S	2022	Y	Y	Y	Y	N	N	Y	Y	Y	N	Y	Y	Y	Y	Y	Y	Moderate
Peng, YN	2020	Y	Y	Y	Y	Y	Y	Y	Y	Y	N	Y	N	N	N	N	Y	Critically low
Naik, A	2022	N	PY	Y	Y	N	N	Y	Y	Y	N	Y	Y	Y	Y	Y	Y	Low
Siccoli, A	2019	N	PY	Y	Y	Y	Y	Y	Y	N	N	Y	N	N	Y	N	N	Critically low
Staartjes, VE	2018	N	Y	Y	PY	N	N	Y	Y	Y	N	Y	Y	Y	Y	Y	N	Low
Tarawneh, AM.	2021	Y	Y	Y	Y	Y	Y	Y	Y	Y	N	Y	Y	N	Y	N	Y	Critically low
Wei, FL	2022	Y	Y	Y	Y	Y	Y	Y	Y	Y	N	Y	Y	Y	Y	N	N	Low
Yu, LJ	2018	N	Y	Y	Y	Y	Y	Y	Y	Y	N	Y	Y	Y	Y	N	N	Low
Zhou, LP	2020	Y	N	N	Y	Y	Y	Y	Y	Y	N	Y	Y	Y	Y	Y	N	Low
Zhou, LP	2021	Y	Y	Y	Y	N	Y	Y	Y	Y	N	Y	Y	Y	Y	Y	Y	Moderate
Zhou, LP	2023	Y	Y	Y	Y	Y	Y	Y	Y	N	N	Y	N	N	Y	N	N	Critically low

*AMSTAR-2 questions.

Q1. Did the research questions and inclusion criteria for the review include the components of PICO? (Yes/No).

Q2. Did the report of the review contain an explicit statement that the review methods were established prior to the conduct of the review and did the report justify any significant deviations from the protocol? (Yes/Partial Yes/No).

Q3. Did the review authors explain their selection of the study designs for inclusion in the review? (Yes/No).

Q4. Did the review authors use a comprehensive literature search strategy? (Yes/Partial Yes/No).

Q5. Did the review authors perform study selection in duplicate? (Yes/No).

Q6. Did the review authors perform data extraction in duplicate? (Yes/No).

Q7. Did the review authors provide a list of excluded studies and justify the exclusions? (Yes/Partial Yes/No).

Q8. Did the review authors describe the included studies in adequate detail? (Yes/Partial Yes/No).

Q9. Did the review authors use a satisfactory technique for assessing the risk of bias (RoB) in individual studies that were included in the review? (Yes/Partial Yes/No).

Q10. Did the review authors report on the sources of funding for the studies included in the review? (Yes/No).

Q11. If meta-analysis was performed did the review authors use appropriate methods for statistical combination of results? (Yes/No).

Q12. If meta-analysis was performed, did the review authors assess the potential impact of RoB in individual studies on the results of the meta-analysis or other evidence synthesis? (Yes/No).

Q13. Did the review authors account for RoB in individual studies when interpreting/ discussing the results of the review? (Yes/No).

Q14. Did the review authors provide a satisfactory explanation for, and discussion of, any heterogeneity observed in the results of the review? (Yes/No).

Q15. If they performed quantitative synthesis did the review authors carry out an adequate investigation of publication bias (small study bias) and discuss its likely impact on the results of the review? (Yes/No).

Q16. Did the review authors report any potential sources of conflict of interest, including any funding they received for conducting the review? (Yes/No).

### Main outcomes

#### Pedicle screw accuracy

**Figure 2.**
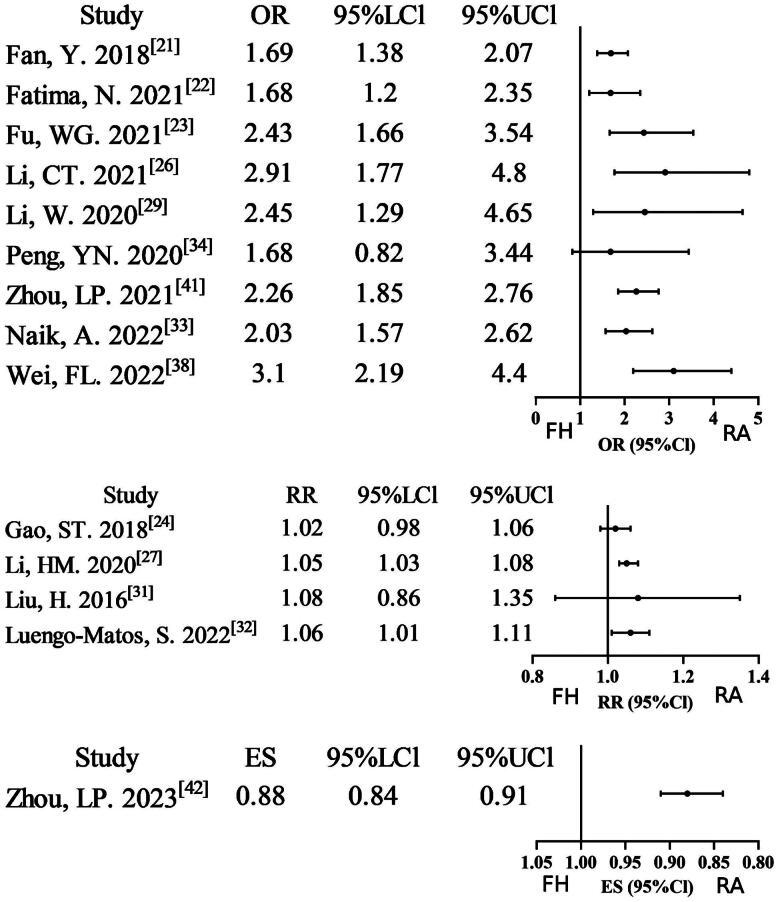
Forest plot of ‘perfect’ pedicle screw (outcome reported as or, RR, and ES).

**Figure 3.**
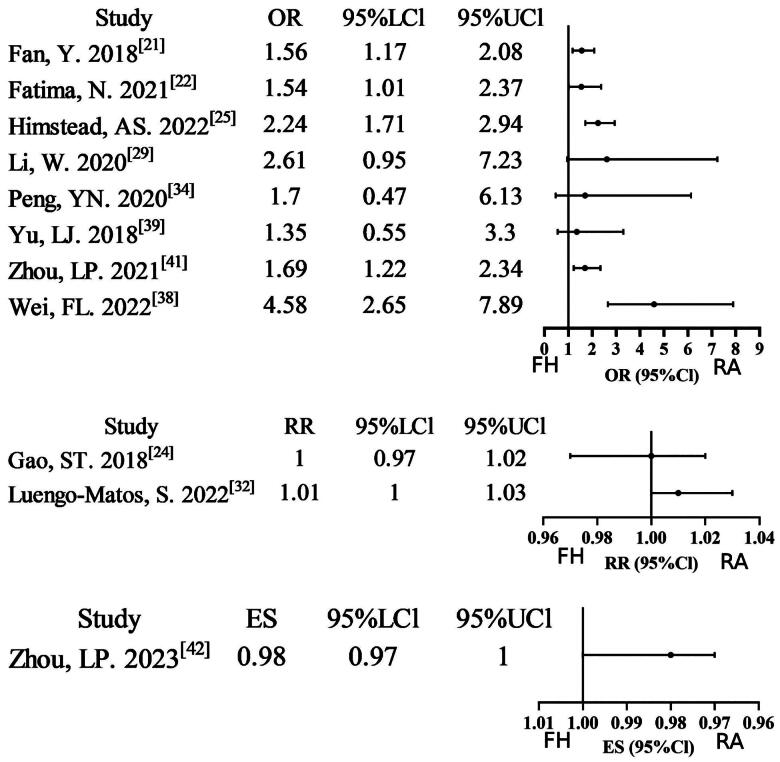
Forest plot of ‘clinically acceptable’ pedicle (outcome reported as or, RR, and ES).

Fifteen meta-analyses [21–24,26, 27,29, 31–34,37, 38,41,42] reported data on pedicle screws that were considered to be ‘perfect’ if they were completely within the pedicle. Twelve studies (12/15, 80%) found a significant improvement in the robot-assisted technique group compared to the free-hand technique group. Two studies were assigned a rating of very low evidence certainty due to the combination of non-RCT/RCT analyses and severe inconsistency. Nine studies were downgraded to low evidence certainty due to non-RCT/RCT merging or severe inconsistency. Three studies exhibiting moderate inconsistency were assigned moderate evidence certainty, while a single study satisfied all methodological standards for high evidence certainty. The GRADE assessment indicates a relatively low level of evidence certainty (Supplementary Table S2; Figure 2). Twelve meta-analyses [21,22, 24,25, 29,32, 34,37–39, 41,42] reported data on the use of ‘clinically acceptable’ pedicle screws. Seven of these studies (7/12, 58.33%) found a significant improvement in the use of ‘clinically acceptable’ pedicle screws in the robot-assisted technique group compared to the free-hand technique group. Three studies were assigned a rating of very low evidence certainty due to the combination of non-RCT/RCT analyses and the presence of inconsistency. Five studies were downgraded to low evidence certainty due to non-RCT/RCT merging or severe inconsistency. Three studies exhibiting moderate inconsistency were assigned moderate evidence certainty, while a single study satisfied all methodological standards for high evidence certainty. Overall, the GRADE assessment indicates relatively low evidence certainty (Supplementary Table S2; Figure 3). A total of two meta-analyses [27,31] reported data on Grade B pedicle screws. Of these, one study (1/2, 50%) found a significant improvement in Grade B pedicle screws in the robot-assisted technique group compared to the free-hand technique group. One study low quality from non-RCT/RCT pooling. The findings of one particular study were of a sufficiently elevated standard to be retained, with no necessity for downgrading. Overall, the GRADE assessment indicates relatively moderate evidence certainty (Supplementary Table S2, Supplementary Figure 1). A total of four meta-analyses [27,34,40] reported data on Grade C + D+E pedicle screws. Of these, two studies (2/4, 50%) found a significant lower in Grade C + D+E pedicle screws in the robot-assisted technique group compared to the free-hand technique group. One study was of low quality due to the non-RCT/RCT pooling. Three studies, which exhibited minor inconsistencies, were assigned a moderate quality rating, while one study, despite its notable limitations, maintained a high quality rating without necessitating a downgrade. Overall, the GRADE assessment indicates relatively moderate evidence certainty (Supplementary Table S2; Supplementary Figure 2).

#### Operative time

**Figure 4.**
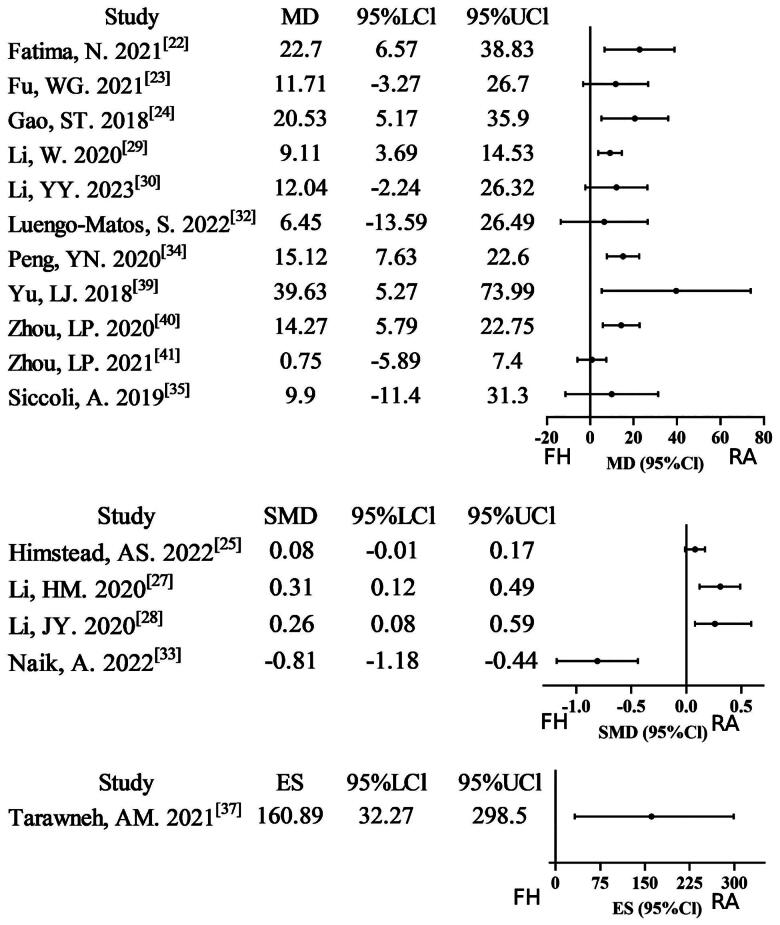
Forest plot of OT (outcome reported as MD, SMD, and ES).

Sixteen meta-analyses [22–25,27–30,32–35,37,39–41], reported data on operative time (OT). Of these, ten studies (10/16, 62.5%) found that the robot-assisted technique group required more time than the free-hand technique group on OT. Six studies were assigned a rating of very low evidence certainty due to combined non-RCT/RCT analyses, severe inconsistency, and imprecision. Five studies were downgraded to low evidence certainty due to non-RCT/RCT merging or severe inconsistency. One study with moderate inconsistency was assigned moderate evidence certainty, while four studies met all methodological standards for high evidence certainty. Overall, the GRADE assessment indicates relatively low evidence certainty (Supplementary Table S2; Figure 4).

#### Length of stay

**Figure 5.**
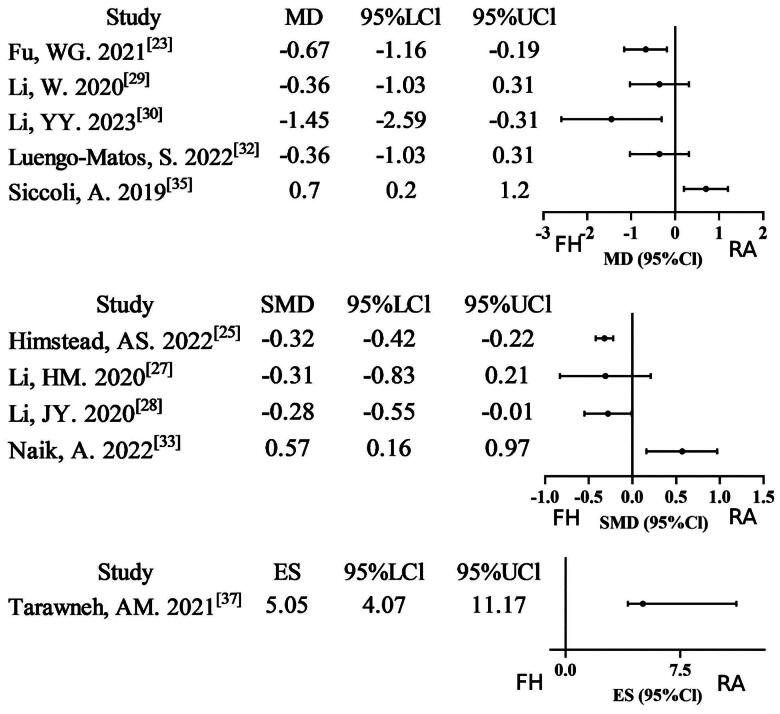
Forest plot of LOS (outcome reported as MD, SMD, and ES).

A total of ten meta-analyses [23,25, 27–30,32, 33,35,37] reported data on length of stay (LOS), and seven studies (7/10, 70%) found that the robot-assisted technique group had lower LOS compared to the free-hand technique group. Four studies were assigned a rating of very low evidence certainty due to the combination of non-RCT/RCT analyses and severe inconsistency. In the course of the study, one piece of research was demoted to low evidence certainty on the basis of non-RCT/RCT merging or severe inconsistency. Four studies exhibiting moderate inconsistency were assigned moderate evidence certainty, while a single study satisfied all methodological standards for high evidence certainty. Overall, the GRADE assessment indicates relatively low evidence certainty (Supplementary Table S2; Figure 5).

#### Intraoperative blood loss

**Figure 6.**
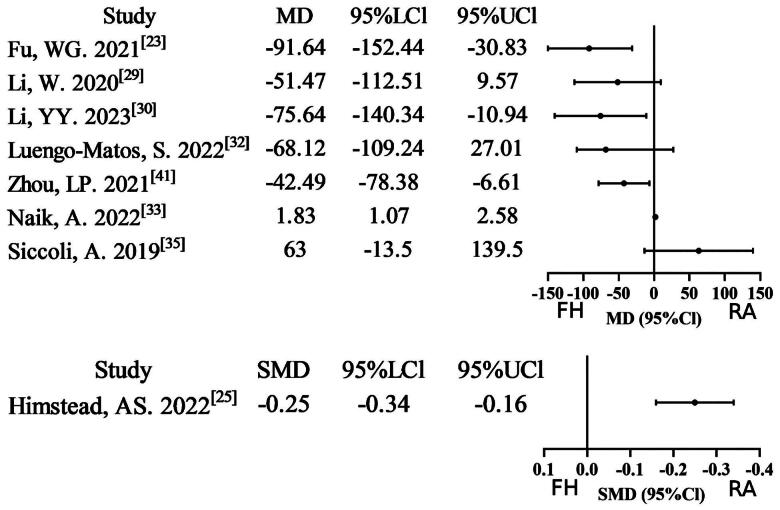
Forest plot of IBL (outcome reported as MD and SMD).

A total of eight meta-analyses [23,25, 29,30, 32,33, 35,41] reported data on intraoperative blood loss (IBL). Of these, five studies (5/8, 62.5%) found that the robot-assisted technique group had lower IBL compared to the free-hand technique group. Five studies were assigned a rating of very low evidence certainty due to combined non-RCT/RCT analyses, severe inconsistency, and severe imprecision. In the course of the present study, three studies were downgraded to low evidence certainty. This occurred due to non-RCT/RCT merging, severe inconsistency, or severe imprecision. Overall, the GRADE assessment indicates relatively very low evidence certainty (Supplementary Table S2; Figure 6).

#### Facet joint violation

**Figure 7.**
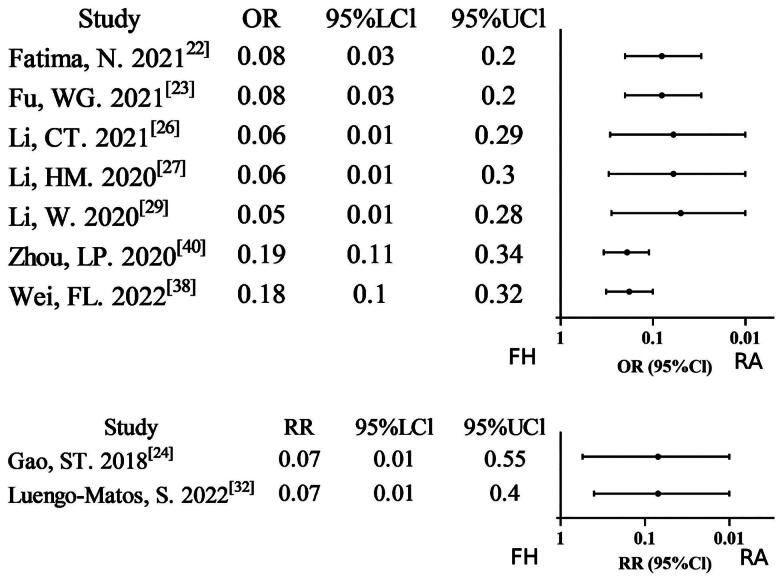
Forest plot of FJV (outcome reported as or and RR).

A total of nine meta-analyses [22–24,26, 27,29, 32,38,40] reported data on facet joint violation (FJV). Of these, nine studies (9/9, 100%) found that the robot-assisted technique group had lower FJV compared to the free-hand technique group. It is evident that three studies were downgraded to low evidence certainty, a consequence of two factors. Firstly, the studies in question were non-RCT/RCT merging. Secondly, the studies exhibited severe inconsistency. Six studies satisfied all methodological standards for high evidence certainty. Overall, the GRADE assessment indicates relatively moderate evidence certainty (Supplementary Table S2; Figure 7).

#### Revision rate

**Figure 8.**
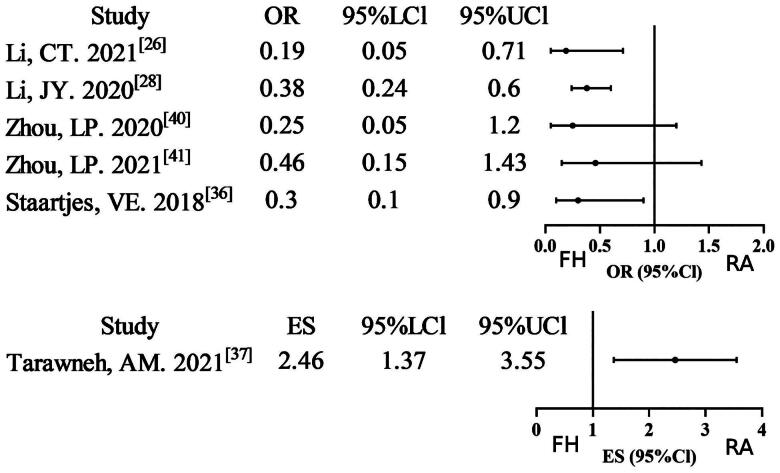
Forest plot of revision rate (outcome reported as or and ES).

Six meta-analyses [26,28, 36,37, 40,41] reported data on the revision rate, and four studies (4/6, 66.67%) found that the robot-assisted technique group had a lower revision rate compared to the free-hand technique group. Five studies were downgraded to low evidence certainty due to non-RCT/RCT merging or severe inconsistency. It is evident that one particular study satisfied all methodological standards for high evidence certainty. Overall, the GRADE assessment indicates relatively low evidence certainty Supplementary Table S2; Figure 8).

### Secondary outcomes

#### Overall complications

A total of eight meta-analyses [22,26, 28,33, 35,38, 39,41] reported data on overall complications. Of these, four studies (4/8, 50%) demonstrated a lower complication rate in robot-assisted technique groups compared to freehand technique groups. The remaining four studies demonstrated no statistically significant differences in total complications between the two groups. Seven studies were downgraded to low evidence certainty due to non-RCT/RCT merging or severe inconsistency. It is evident that one particular study satisfied all methodological standards, thereby ensuring a high level of evidence certainty. Overall, the GRADE assessment indicates relatively low evidence certainty (Supplementary Table S2, Supplementary Figure 3).

#### Intraoperative radiation time

Eleven meta-analyses [22,24, 26–29,32, 34,37–39] reported data on intraoperative radiation time (IRT). Five of these studies (5/11, 45.45%) found that the robot-assisted technique group was superior to the free-hand technique group in terms of IRT. Five studies were assigned a rating of very low evidence certainty due to combined non-RCT/RCT analyses, severe inconsistency, and imprecision. Four studies were downgraded to low evidence certainty due to severe inconsistency. One study with moderate inconsistency was assigned moderate evidence certainty, while one study satisfied all methodological standards for high evidence certainty. Overall, the GRADE assessment indicates relatively very low evidence certainty (Supplementary Table S2, Supplementary Figure 4).

#### Intraoperative radiation dose

Eleven meta-analyses [22–24,27–29,32,35,37,38,40], reported data on intraoperative radiation dose (IRD). Of these, nine studies (9/11, 81.82%) found that the robot-assisted technique group was superior to the free-hand technique group with regard to IRD. Five studies were assigned a rating of very low evidence certainty due to the combination of non-RCT/RCT analyses and severe inconsistency. It is evident that three studies downgraded to low evidence certainty as a consequence of severe inconsistency. One study with moderate inconsistency was assigned moderate evidence certainty, while two studies satisfied all methodological standards for high evidence certainty. Overall, the GRADE assessment indicates relatively very low evidence certainty (Supplementary Table S2; Supplementary Figure 5).

#### Visual analogue scale

Six meta-analyses [23,27, 29,30, 33,37] reported data on the visual analogue scale (VAS). However, no studies found that the robot-assisted technique group was superior to the free-hand technique group on VAS. One study was assigned a rating of very low evidence certainty, primarily due to the integration of non-RCT/RCT analyses, in conjunction with moderate inconsistency. It is evident that the downgrading of one study to low evidence certainty was due to the combination of non-RCT/RCT analyses. Four studies satisfied all methodological standards for high evidence certainty. Overall, the GRADE assessment indicates relatively moderate evidence certainty (Supplementary Table S2; Supplementary Figure 6).

#### Oswestry disability index

A total of five meta-analyses [23,27, 29,30,33] reported data on the Oswestry Disability Index (ODI). However, no studies were found that indicated the robot-assisted technique group was superior to the free-hand technique group on the ODI. One study was assigned a rating of very low evidence certainty, primarily due to the combination of non-RCT/RCT analyses, in conjunction with moderate inconsistency. Two studies were downgraded to low evidence certainty due to combined non-RCT/RCT analyses or severe inconsistency. Two studies satisfied all methodological standards for high evidence certainty. Overall, the GRADE assessment indicates relatively low evidence certainty (Supplementary Table S2; Supplementary Figure 7).

## Discussion

This umbrella review systematically evaluated 22 meta-analyses encompassing RCTs, cohort studies, and observational studies comparing robot-assisted and freehand techniques in spine surgery. The GRADE assessment revealed that the majority of outcomes – including pedicle screw accuracy, operative time, intraoperative radiation exposure, and so on – were supported by low to very low certainty of evidence. These findings emphasize the necessity for a meticulous examination of the extant meta-analytical evidence.

The utilization of robotic-assisted techniques in spine surgery has been demonstrated to offer a number of potential benefits. The findings from the meta-analyses demonstrated consistent advantages of robot-assisted techniques in improving pedicle screw placement precision (‘perfect’ and ‘clinically acceptable’ screw rates) and reducing FJV [43]. The findings of the present study indicated that all meta-analyses addressing FJV (9/9, 100%) demonstrated reduced violation rates and GRADE assessment was supported by moderate certainty of evidence. These outcomes align with biomechanical principles suggesting that robotic systems enhance surgical precision through preoperative planning and intraoperative guidance. Li et al. [44]. conducted a systematic analysis which demonstrated that robot-assisted screw placement significantly reduced the rate of proximal facet joint violation (FJV), with an incidence of 4% compared to 26% in freehand placement (OR = 0.31, *p* < 0.001). Furthermore, the severity of FJV (graded 0–3) was significantly improved (Mean Grade: 0.05 vs. 0.38). Similarly, Yu et al. [38]. conducted a network meta-analysis, which further validated that robotic system such as the Renaissance and TiRobot, outperformed traditional freehand techniques in reducing FJV (OR = 0.28–0.42). This outcome was attributed to the precision of preoperative planning and mechanical guidance. It is evident that FJV has been a critical concern, and this umbrella review summarizes existing evidence, further highlighting the advantages of robot-assisted spinal surgery in minimizing FJV. The merits of FJV mitigation are more evidently manifest in the reduction of adjacent segment degeneration (ASD). Robotic technology provides real-time navigation and precise intraoperative planning, thereby optimizing surgical trajectories and minimizing biomechanical stress alterations caused by surgical interventions [45]. A long-term follow-up study by Li et al. [46]. demonstrated that robot-assisted pedicle screw placement reduces the risk of ASD by preserving mobile segments (e.g. decreasing the need for adjacent segment decompression). Concurrently, research by Chen et al. [47]. revealed that the enhanced accuracy of robotic screw placement mitigates biomechanical changes associated with rigid fixation in fused segments, thereby delaying the progression of ASD.

While computer-assisted navigation systems have been shown to achieve enhanced accuracy in instrumented spine procedures for elective indications such as degenerative pathologies and deformity corrections, the comparative efficacy of robotic platforms in achieving superior precision remains to be validated through prospective comparative trials. Robotic-assisted spinal systems are dependent on preoperative CT imaging for trajectory planning; however, they are unable to provide real-time intraoperative guidance [7]. During screw placement, surgeons must still rely on fluoroscopy for dynamic adjustments, as it remains the gold standard for real-time feedback. While haptic feedback in robotic systems has been demonstrated to effectively minimize manual tremor during instrumentation [48,49], it has not been shown to compensate for the absence of intraoperative imaging [50]. Notwithstanding the aforementioned limitations, robotic assistance facilitates minimally invasive approaches in complex cases. Advancements in imaging technologies, such as high-resolution CT and MRI, have further enhanced anatomical visualization for the purpose of preoperative planning [51]. The findings of our analysis demonstrate the clinical advantages of robotic assistance, which have a direct impact on patient care. A 2023 study by Neurospine demonstrated that the accuracy of robot-assisted screw placement accuracy, utilizing systems such as the ExcelsiusGPS, reached 96.4%, significantly surpasses the 92% achieved with the traditional freehand technique [52]. The findings of the present study indicated that, with regard to ‘perfect’ screw placement, 12/15 studies (80%) demonstrated superior performance in the robot-assisted group in comparison to the freehand group. Furthermore, over half of the studies (7/12, 58.33%) reported greater advantages for the robot-assisted group in achieving ‘clinically acceptable’ screw placement. However, the pooled effect sizes for these outcomes were consistently associated with significant heterogeneity and methodological limitations in primary studies, resulting in reduced certainty of evidence.

A study also revealed that the complication rate for robot-assisted screws was only 2.8%, with only one case requiring revision [52]. The research conducted by Ueno et al. [53]. on robot-assisted cortical bone trajectory (CBT) screws demonstrated an exceptional accuracy rate of up to 95.3%. Furthermore, the absolute increase in clinically acceptable screw placement was associated with a 40% reduction in revision surgery rates among the studies included. These findings are consistent with the 2023 AO Spine clinical guidelines, which define a ‘clinically meaningful reduction’ in the risk of reoperation, with the defined threshold being met. This finding is consistent with the results of the present study, which demonstrated that half of the studies assessing overall complication rates (4/8, 50%) exhibited a lower incidence in robot-assisted technique groups compared to freehand technique groups. Furthermore, a higher proportion of studies (4/6, 66.67%) reported a lower revision rate in the robot-assisted group. Despite being assigned a low level of certainty of evidence by the GRADE framework, these findings nevertheless possess a certain degree of clinical relevance in terms of guiding current practice. From a clinical perspective, the observed decline in revision rates (with 66.7% of studies supporting the utilization of robotics) and the reduced risk of ASD may result in long-term cost savings. However, economic analyses are absent in the majority of the included studies, and the initial costs of robotic systems pose significant barriers to widespread adoption. Furthermore, the employment of robotic systems has been demonstrated to reduce radiation exposure for both patients and surgical teams [54,55]. It is noteworthy that the results of the studies are not consistent, with some studies reporting a prolonged operative time with robotics (62.5%), and others showing a reduced radiation exposure with robotics (81.8%), although 45.5% of these studies did not report any difference in time. For instance, percutaneous C1–C2 posterior instrumentation requires real-time anatomical adaptability beyond the current capabilities of robotic systems, as demonstrated by Farah et al. [55]. who noted frequent intraoperative navigation adjustments even with the assistance of a robotic system. Furthermore, the utilization of robotic systems in emergency scenarios such as acute spinal trauma accompanied by neurological compromise, is less optimal. The technical setup time and reliance on preoperative imaging limit adaptability to dynamic intraoperative changes inherent in trauma cases. It is important to note that none of the included studies addressed emergent indications, which serves to reinforce the need for cautious extrapolation of current findings to urgent clinical settings. It is evident that robotic-assisted techniques in spine surgery represent a substantial advancement, offering to propose minimal invasive surgery and reproducibility. However, both robotic-assisted and free-hand techniques have their distinct advantages and limitations in terms of spine surgery.

While robotic-assisted systems have been demonstrated to exhibit clear advantages in terms of screw placement accuracy and radiation reduction, their adoption is tempered by significant practical limitations. The existence of high capital costs and recurrent maintenance expenses has been demonstrated to act as significant barriers to the widespread implementation of the system, particularly healthcare systems that are characterized by limited resources [56]. Furthermore, the acquisition of proficiency in robotic platforms necessitates a considerable learning curve, with proficiency typically requiring approximately 50 cases based on prior competency assessments [57,58]. Technical failures or malfunctions in robotic systems are a possibility, with the potential to necessitate additional time and resources to resolve [59]. Furthermore, the robot-assisted technique group required a greater duration to free-hand the technique group on OT, a factor which should be given full consideration in light of operating room occupancy duration, encompassing patient positioning and robotic system setup. These contributing elements may be responsible potentially account for the observed increase in surgical timelines.

The freehand technique has been demonstrated to offer distinct advantages in clinical practice. The cost-effectiveness of the system is primarily driven by the absence of reliance on expensive robotic systems and associated maintenance [60,61]. The surgeons also benefit from procedural flexibility, as freehand methods allow intraoperative adjustments unconstrained by the spatial limitations of robotic platforms [62,63]. Furthermore, the extensive familiarity of spine surgeons with freehand techniques has the potential to enhance workflow efficiency and reduce procedural complexity, particularly in settings with limited robotic training resources [64]. Nevertheless, these advantages are mitigated by critical limitations. Freehand screw placement has been demonstrated to demonstrate a reduced level of accuracy in comparison to robotic-assisted methods, especially in anatomically challenging regions such as the upper thoracic spine or revision surgeries [30,65]. Finally, the utilization of fluoroscopy has been demonstrated to engender elevated levels of radiation exposure for both patients and surgical teams, thereby giving rise to long-term safety concerns [66,67].

Whilst the present umbrella review concentrated on the comparison of robot-assisted and freehand techniques, it is recognized that there is a significant clinical need to evaluate robot-assisted systems in comparison with navigation-assisted modalities. The latter represents a mature technology that has been widely adopted in spinal instrumentation. However, comparative meta-analyses between these two advanced paradigms remain scarce, reflecting a critical gap in high-level evidence. Subsequent umbrella reviews should accord this comparison a higher priority as soon as a sufficient number of systematic reviews and RCTs have been accumulated. The present review is the first to systematically assess the strength of evidence and clinical effects of free-hand versus robotic technique by analyzing published systematic reviews and meta-analyses. However, the present review did not address the distinction between preoperative CT-based robotic navigation and intraoperative fluoroscopic guidance. Robot-assisted systems are deficient in true real-time imaging, which may limit adaptability to intraoperative anatomical shifts in comparison with fluoroscopy. As with any umbrella review, there are certain limitations to the study that may have an impact on its overall quality. Firstly, the inclusion criteria of the included meta-analyses and systematic reviews varied; some focused exclusively on RCTs, while others included both RCTs and non-RCTs. The incorporation of non-RCTs has the potential to introduce biases and yield less robust outcomes. Consequently, the findings from non-RCTs should be interpreted with caution when applied to clinical practice. Secondly, the reliability of the conclusions is contingent upon the quality of the meta-analyses and systematic reviews incorporated within the review, which may consequently result in limitations being imposed on the findings. Thirdly, as the GRADE assessment system and AMSTAR2 were utilized to evaluate the pooled studies, it was found that the methodological quality of the majority of the included meta-analyses and systematic reviews was rated as moderate to very low. Finally, methodological constraints, the collective evidence suggest that robotic systems may reduce reoperation risks and ASD by reducing biomechanical stress through precise screw placement. However, it is imperative to consider the economic and practical limitations that may hinder the translation of these benefits into clinical practice, including significant costs and the necessity for extensive technical training.

## Conclusion

This systematic review demonstrates a significant correlation between robot-assisted spinal surgery and favorable clinical outcomes. Specifically, compared to conventional freehand technique, the robot-assisted technique demonstrates superior pedicle screw placement precision, reduced revision rates, and lower incidence of FJV. Furthermore, although robot-assisted procedures require a longer operative duration, they demonstrate clinically meaningful advantages including decreased IRD and IRT, shortened LOS, and reduced IBL. However, given the low certainty of the evidence, clinicians should interpret our results with caution when applying them in clinical practice, and high-quality studies are required in the future to confirm our results.

## Research registration information

This systematic review was performed according to Preferred Reporting Items for Systematic Reviews and Meta-Analysis guidelines and was registered at PROSPERO (CRD42023471380).

## Ethics approval and consent to participate

Meta-analyses do not involve human subjects and do not require IRB review (J Grad Med Educ. 2011 March; 3 (1): 5–6).

## Clinical trial number

Not applicable

## Consent for publication

Not applicable

## Supplementary Material

PRISMA2020checklist.docx

Supplementary_Table_S1 - Clean.docx

Supplementary Table S2.docx

## Data Availability

All data generated or analyzed during this study are included in this published article [and its supplementary information files]

## Abbreviations

PRISMA: Preferred Reporting Items for Systematic Reviews and Meta-Analysis
AMSTAR-2: A Measurement Tool to Assess Systematic Reviews
GRADE: Grading of Recommendations, Assessment, Development, and Evaluations
RCTs: Randomized Controlled Trials
PKP: Percutaneous Kyphoplasty
PVP: Percutaneous Vertebroplasty
FJV: Facet Joint Violation
IRD: Intraoperative Radiation Dose
IRT: Intraoperative Radiation Time
OT: Operative Time
LOS: Length of Stay
IBL: Intraoperative Blood Loss
VAS: Visual Analogue Scale
ODI: Oswestry disability index
