# Supplementary material for: Robot-assisted technique versus freehand technique for spine surgery: an umbrella review
Source: Ann Med. 2025 Jul 9;57(1):2523564. doi: 10.1080/07853890.2025.2523564 (PMC12243020; doi:10.1080/07853890.2025.2523564)
Supplement: Supplementary_Table_S1 - Clean.docx [file IANN_A_2523564_SM8686.docx]

Supplementary Table S1: Index and Keyword Terms Used in the Databases.

| Databases | Search strategy |
| --- | --- |
| PubMed: | ((((((((robot-assisted surgery[Title/Abstract]) OR (robot-assisted[Title/Abstract])) OR (robot[Title/Abstract])) OR (robotic[Title/Abstract])) OR (robot assisted surgery[Title/Abstract])) OR (robot-assisted surgeries[Title/Abstract])) OR (Robotic Surgical Procedures[Title/Abstract])) OR ("Robotic Surgical Procedures"[Mesh])) AND (((((((((((((Vertebral Column[Title/Abstract]) OR (Column, Vertebral[Title/Abstract])) OR (Columns, Vertebral[Title/Abstract])) OR (Vertebral Columns[Title/Abstract])) OR (Spinal Column[Title/Abstract])) OR (Column, Spinal[Title/Abstract])) OR (Columns, Spinal[Title/Abstract])) OR (Spinal Columns[Title/Abstract])) OR (Vertebra[Title/Abstract])) OR (Vertebrae[Title/Abstract])) OR (spine[Title/Abstract])) OR (spinal[Title/Abstract])) OR ("Spine"[Mesh])) AND (meta-analysis[Filter]) |
| Cochrane database | #1 Mesh descriptor: [Spine] explode all trees  #2 (Vertebral Column): ti, ab, kw OR (Column, Vertebral): ti, ab, kw OR (Columns, Vertebral): ti, ab, kw OR (Vertebral Columns): ti, ab, kw OR (Spinal Column): ti, ab, kw OR (Column, Spinal): ti, ab, kw OR (Spinal Columns): ti, ab, kw OR (Spinal Columns): ti, ab, kw OR (Vertebra): ti, ab, kw OR (Vertebrae): ti, ab, kw  #3 #1 OR #2  #4 Mesh descriptor: [Robotic Surgical Procedures] explode all trees  #5 (robot-assisted surgery): ti, ab, kw OR (robot-assisted): ti, ab, kw OR (robot): ti, ab, kw OR (robotic): ti, ab, kw OR (robot assisted surgery): ti, ab, kw OR (robot-assisted surgeries): ti, ab, kw OR (Robotic Surgical Procedures): ti, ab, kw  #6 #4 OR #5  #7 (meta-analysis): ti, ab, kw  #8 #3 and #6 and #7 |
| Embase | #1 “Spine”/exp OR “Spine”  #2 “Vertebral Column”: ti, ab, kw OR “Column, Vertebral”: ti, ab, kw OR “Columns, Vertebral”: ti, ab, kw OR “Vertebral Columns”: ti, ab, kw OR “Spinal Column”: ti, ab, kw OR “Column, Spinal”: ti, ab, kw OR “Spinal Columns”: ti, ab, kw OR “Spinal Columns”: ti, ab, kw OR “Vertebra”: ti, ab, kw OR “Vertebrae”: ti, ab, kw  #3 #1 OR #2  #4 “Robot assisted surgery”/exp OR “Robot assisted surgery”  #5 “robot-assisted surgery”: ti, ab, kw OR “robot-assisted”: ti, ab, kw OR “robot”: ti, ab, kw OR “robotic”: ti, ab, kw OR “robot assisted surgery”: ti, ab, kw OR “robot-assisted surgeries”: ti, ab, kw OR “Robotic Surgical Procedures”: ti, ab, kw  #6 #4 OR #5  #7 “meta-analysis”: ti, ab, kw  #8 #3 and #6 and #7 |
| Web of Science | TS=(Robotic Surgical Procedures or Robotic Surgical Procedures or robot-assisted surgeries or robot assisted surgery or robotic or robot or robot-assisted or robot-assisted surgery) and TS=(Vertebral Column or Column, Vertebral or Columns, Vertebral or Vertebral Columns or Spinal Column or Column, Spinal or Columns, Spinal or Spinal Columns or Vertebra or Vertebrae or spine or spinal) and TS=(meta-analysis) |
| Scopus | KEY("Robotic Surgical Procedures" or "Robotic Surgical Procedures" or "robot-assisted surgeries" or "robot assisted surgery" or "robotic" or "robot or robot-assisted" or "robot-assisted surgery") and KEY("Vertebral Column" or "Column, Vertebral" or "Columns, Vertebral" or "Vertebral Columns" or "Spinal Column" or "Column, Spinal" or "Columns, Spinal" or "Spinal Columns" or "Vertebra" or "Vertebrae" or "spine" or "spinal") and KEY("meta-analysis") |
