# Supplementary material for: Robot-assisted technique versus freehand technique for spine surgery: an umbrella review
Source: Ann Med. 2025 Jul 9;57(1):2523564. doi: 10.1080/07853890.2025.2523564 (PMC12243020; doi:10.1080/07853890.2025.2523564)
Supplement: Supplementary Table S2.docx [file IANN_A_2523564_SM8685.docx]

| Supplementary Table S2: Characteristics and certainty of evidence estimates for the main outcome | | | | | |
| --- | --- | --- | --- | --- | --- |
| Outcome Author,year | Relative effect (95%CI) | I-squared | I-squared P-value | Certainty | Combined Certainty |
| **“Perfect” pedicle screw** | **OR (95%CI)** |  |  |  |  |
| Fan, Y. 2018^[21]^ | 1.69(1.38-2.07) | 82% | <0.0001 | Very Low ^b,g^ | Low |
| Fatima, N. 2021^[22]^ | 1.68(1.20-2.35) | 77% | <0.0001 | Very Low ^b,g^ |  |
| Fu, WG. 2021^[23]^ | 2.43(1.66-3.54) | 68% | <0.0001 | Moderate ^a,d^ |  |
| Li, CT. 2021^[26]^ | 2.91(1.77-4.80) | 76% | <0.0001 | Low ^a,g^ |  |
| Li, W. 2020^[29]^ | 2.45(1.29-4.65) | 77% | <0.0001 | Low ^a,g^ |  |
| Peng, YN. 2020^[34]^ | 1.68(0.82-3.44) | 77% | 0.0002 | Low ^a,g^ |  |
| Zhou, LP. 2021^[41]^ | 2.26(1.85-2.76) | 37% | 0.16 | Low ^b^ |  |
| Naik, A. 2022^[33]^ | 2.03(1.57-2.62) | - | - | Low ^b^ |  |
| Wei, FL. 2022^[38]^ | 3.10(2.19-4.40) | - | - | Low ^b^ |  |
|  | **RR (95%CI)** |  |  |  |  |
| Gao, ST. 2018^[24]^ | 1.02(0.98-1.06) | 51% | 0.07 | Moderate ^a,d^ |  |
| Li, HM. 2020^[27]^ | 1.05(1.03-1.08) | 54% | 0.04 | Moderate ^a,d^ |  |
| Liu, H. 2016^[31]^ | 1.08(0.86-1.35) | 28% | 0.23 | Low ^b^ |  |
| Luengo-Matos, S. 2022^[32]^ | 1.06(1.01-1.11) | 87% | <0.0001 | Low ^a,g^ |  |
|  | **ES (95%CI)** |  |  |  |  |
| Tarawneh, AM. 2021^[37]^ | 84.22(22.38-146.05) | 0% | 0.952 | High ^a^ |  |
| Zhou, LP. 2023^[42]^ | 0.88(0.84-0.91) | 47.94% | 0.073 | Low ^b^ |  |
|  |  |  |  |  |  |
| **“Clinically acceptable” pedicle screw** | **OR (95%CI)** |  |  |  |  |
| Fan, Y. 2018^[21]^ | 1.56(1.17-2.08) | 55% | 0.02 | Very Low ^b,g^ | Low |
| Fatima, N. 2021^[22]^ | 1.54(1.01-2.37) | 61% | <0.0001 | Very Low ^b,d^ |  |
| Himstead, AS. 2022^[25]^ | 2.24(1.71-2.94) | 16% | 0.2 | Low ^b^ |  |
| Li, W. 2020^[29]^ | 2.61(0.95-7.23) | 64% | 0.005 | Moderate ^a,d^ |  |
| Peng, YN. 2020^[34]^ | 1.70(0.47-6.13) | 71% | 0.002 | Moderate ^a,d^ |  |
| Yu, LJ. 2018^[39]^ | 1.35(0.55-3.30) | 70% | 0.001 | Very Low ^b,d^ |  |
| Zhou, LP. 2021^[41]^ | 1.69(1.22-2.34) | 0% | 0.93 | Low ^b^ |  |
| Wei, FL. 2022^[38]^ | 4.58(2.65-7.89) | - | - | Low ^b^ |  |
|  | **RR (95%CI)** |  |  |  |  |
| Gao, ST. 2018^[24]^ | 1.00(0.97-1.02) | 73% | 0.002 | Moderate ^a,d^ |  |
| Luengo-Matos, S. 2022^[32]^ | 1.01(1.00-1.03) | 81% | <0.0001 | Low ^a,g^ |  |
|  | **ES (95%CI)** |  |  |  |  |
| Tarawneh, AM. 2021^[37]^ | 86.96(21.54-152.36) | 0% | 0.948 | High ^a^ |  |
| Zhou, LP. 2023^[42]^ | 0.98(0.97-1.00) | 35.95% | 0.167 | Low ^b^ |  |
|  |  |  |  |  |  |
| **Grade B pedicle screw** | **RR (95%CI)** |  |  |  |  |
| Li, HM. 2020^[27]^ | 0.70(0.52-0.94) | 29% | 0.21 | High ^a^ | Moderate |
| Liu, H. 2016^[31]^ | 1.02(0.68-1.51) | 0.46 | 0.12 | Low ^b^ |  |
|  |  |  |  |  |  |
| **Grade C+D+E pedicle screw** | **OR (95%CI)** |  |  |  |  |
| Peng, YN. 2020^[34]^ | 0.59(0.16-2.12) | 71% | 0.002 | Moderate ^a,d^ | Moderate |
| Zhou, LP. 2020^[40]^ | 0.24(0.14-0.43) | 0% | - | Low ^b^ |  |
|  | **RR (95%CI)** |  |  |  |  |
| Li, HM. 2020^[27]^ | 0.52(0.33-0.83) | 66% | 0.008 | Moderate ^a,d^ |  |
|  | **ES (95%CI)** |  |  |  |  |
| Tarawneh, AM. 2021^[37]^ | 0.53(-0.78-1.83) | 9.3% | 0.353 | High ^a^ |  |

Notes: a, All RCTs Meta-analysis (GRADE assessment begins with High evidence); b, RCTs and None-RCTs Meta-analysis (GRADE assessment begins with Low evidence); c, Risk of bias (going from assessments of risk of bias to judgments about study limitations for main outcomes across all included studies); d, Inconsistency (small P value from a test for heterogeneity or a high I^2^ value, 50%＜I^2^≤75%); e, Indirectness (differences in population, intervention and outcome measures); f, Imprecision (wide 95% confidence interval around the estimate of the effect); g, Severe inconsistency（75%＜I^2^）; h, Severe imprecision (very wide 95% confidence interval around the estimate of the effect)

**Secondary outcomes**

| **Characteristics and certainty of evidence estimates for the clinical date** | | | | | |
| --- | --- | --- | --- | --- | --- |
| Outcome Author, year | Relative effect (95%CI) | I-squared | I-squared P-value | Certainty | Combined Certainty |
| **Overall complications** | **OR (95%CI)** |  |  |  |  |
| Fatima, N. 2021^[22]^ | 0.31(0.20-0.48) | 9% | 0.36 | Low ^b^ | Low |
| Li, CT. 2021^[26]^ | 0.39(0.10-1.48) | 0% | 0.97 | High ^a^ |  |
| Li, JY. 2020^[28]^ | 0.57(0.28-1.15) | 0% | 0.70 | Low ^b^ |  |
| Yu, LJ. 2018^[39]^ | 0.46(0.15-1.43) | 0% | 0.69 | Low ^b^ |  |
| Zhou, LP. 2021^[41]^ | 0.65(0.32-1.33) | 0% | - | Low ^b^ |  |
| Naik, A. 2022^[33]^ | 0.40(0.24-0.68) | - | - | Low ^b^ |  |
| Siccoli, A. 2019^[35]^ | 1.60(1.30-1.90) | - | - | Low ^b^ |  |
| Wei, FL. 2022^[38]^ | 0.38(0.23-0.63) | 27.05% | 0.26 | Low ^b^ |  |
|  |  |  |  |  |  |
| **FJV** | **OR (95%CI)** |  |  |  |  |
| Fatima, N. 2021^22]^ | 0.08(0.03-0.20) | 0% | 0.70 | Low ^b^ | Moderate |
| Fu, WG. 2021^[23]^ | 0.08(0.03-0.20) | 0% | 0.64 | High ^a^ |  |
| Li, CT. 2021^[26]^ | 0.06(0.01-0.29) | 0% | 0.97 | High ^a^ |  |
| Li, HM. 2020^[27]^ | 0.06(0.01-0.30) | 0% | 0.52 | High ^a^ |  |
| Li, W. 2020^[29]^ | 0.05(0.01-0.28) | 0% | 0.48 | High ^a^ |  |
| Zhou, LP. 2020^[40]^ | 0.19(0.11-0.34) | 0% | 0.48 | Low ^b^ |  |
| Wei, FL. 2022^[38]^ | 0.18(0.1-0.32) | 9.55% | 0.37 | Low ^b^ |  |
|  | **RR (95%CI)** |  |  |  |  |
| Gao, ST. 2018^[24]^ | 0.07(0.01-0.55) | 10% | 0.29 | High ^a^ |  |
| Luengo-Matos, S. 2022^[32]^ | 0.07(0.01-0.4) | 0% | - | High ^a^ |  |
|  |  |  |  |  |  |
| **IRT** | **MD (95%CI)** |  |  |  |  |
| Fatima, N. 2021^[22]^ | -5.30(-6.83- -3.76) | 98% | <0.0001 | Very Low ^b,g^ | Very Low |
| Gao, ST. 2018^[24]^ | -12.38(-17.95- -6.80) | 84% | 0.01 | Low ^a,g^ |  |
| Li, CT. 2021^[26]^ | -9.74(-20.18-4.30) | 99% | <0.0001 | Low ^a,g^ |  |
| Li, W. 2020^[29]^ | -2.80(-27.81-22.21) | 98% | <0.0001 | Very Low ^a,g,f^ |  |
| Luengo-Matos, S. 2022^[32]^ | -3.00(-28.01-22.00) | 93% | <0.0001 | Low ^a,g^ |  |
| Peng, YN. 2020^[34]^ | -12.36(-17.92- -6.81) | 84% | 0.01 | Low ^a,g^ |  |
| Yu, LJ. 2018^[39]^ | 0.18(-22.73-23.09) | 99% | <0.0001 | Very Low ^b,g,f^ |  |
| Wei, FL. 2022^[38]^ | 2.45(-10.61-15.51) | 99.51% | < 0.0001 | Very Low^b,g,^ |  |
|  | **SMD (95%CI)** |  |  |  |  |
| Li, HM. 2020^[27]^ | -1.33(-1.77- -0.89) | 67% | 0.08 | Moderate ^a,d^ |  |
| Li, JY. 2020^[28]^ | -0.05(-0.68-0.58) | 98.8% | <0.0001 | Very Low ^b,g^ |  |
|  | **SE (95%CI)** |  |  |  |  |
| Tarawneh, AM. 2021^[37]^ | 14.13(7.43-20.83) | 19.2% | 0.266 | High ^a^ |  |
|  |  |  |  |  |  |
| **IRD** | **MD (95%CI)** |  |  |  |  |
| Fatima, N. 2021^[22]^ | -3.70(-4.80- -2.60) | 99% | <0.0001 | Very Low ^b,g^ | Very Low |
| Fu, WG. 2021^[23]^ | -23.52(-40.12- -6.93) | 96% | <0.0001 | Low ^a,g^ |  |
| Zhou, LP. 2020^[40]^ | -39.23(-53.86- -24.60) | 87% | <0.0001 | Very Low ^b,g^ |  |
| Siccoli, A. 2019^[35]^ | -3.10(-41.60-35.49) | 97% | < 0.0001 | Very Low ^b,g^ |  |
| Wei, FL. 2022^[38]^ | -14.38(-25.62- -3.13) | 100% | < 0.0001 | Very Low ^b,g^ |  |
|  | **SMD (95%CI)** |  |  |  |  |
| Gao, ST. 2018^[24]^ | -0.64(-0.85- -0.43) | 0% | 0.90 | High ^a^ |  |
| Li, HM. 2020^[27]^ | -0.58(-0.79- -0.37) | 0% | 0.89 | High ^a^ |  |
| Li, JY. 2020^[28]^ | -0.55(-1.23-0.13) | 99.1% | <0.0001 | Very Low ^b,g^ |  |
| Li, W. 2020^[29]^ | -1.30(-2.01- -0.60) | 97% | <0.0001 | Low ^a,g^ |  |
| Luengo-Matos, S. 2022^[32]^ | -1.31(-2.02- -0.60) | 87% | <0.0001 | Low ^a,g^ |  |
|  | **SE (95%CI)** |  |  |  |  |
| Tarawneh, AM. 2021^[37]^ | 0.27(0.02-0.53) | 64.9% | 0.091 | Moderate ^a,d^ |  |
|  |  |  |  |  |  |
| **OT** | **MD (95%CI)** |  |  |  |  |
| Fatima, N. 2021^[22]^ | 22.70(6.57-38.83) | 89% | <0.0001 | Very Low ^b,g^ | Low |
| Fu, WG. 2021^[23]^ | 11.71(-3.27-26.70) | 85% | <0.0001 | Low ^a,g^ |  |
| Gao, ST. 2018^[24]^ | 20.53(5.17-35.90) | 10% | 0.33 | High ^a^ |  |
| Li, W. 2020^[29]^ | 9.11(3.69-14.53) | 39% | 0.12 | High ^a^ |  |
| Li, YY. 2023^[30]^ | 12.04(-2.24-26.32) | 98% | <0.0001 | Very Low ^b,g^ |  |
| Luengo-Matos, S. 2022^[32]^ | 6.45(-13.59-26.49) | 74% | 0.004 | Moderate ^a,d^ |  |
| Peng, YN. 2020^[34]^ | 15.12(7.63-22.60) | 78% | 0.0003 | Low ^a,g^ |  |
| Yu, LJ. 2018^[39]^ | 39.63(5.27-73.99) | 88% | 0.0002 | Very Low^b,g^ |  |
| Zhou, LP. 2020^[40]^ | 14.27(5.79-22.75) | 0% | - | Low ^b^ |  |
| Zhou, LP. 2021^[41]^ | 0.75(-5.89-7.40) | 36% | - | Low ^b^ |  |
| Siccoli, A. 2019^[35]^ | 9.90(-11.4-31.30) | 96% | < 0.0001 | Very Low^b,g,f^ |  |
|  | **SMD (95%CI)** |  |  |  |  |
| Himstead, AS. 2022^[25]^ | 0.08(-0.01-0.17) | 88% | <0.01 | Very Low ^b,g^ |  |
| Li, HM. 2020^[27]^ | 0.31(0.12-0.49) | 1% | 0.4 | High ^a^ |  |
| Li, JY. 2020^[28]^ | 0.26(0.08-0.59) | 89.9% | <0.0001 | Very Low ^b,g^ |  |
| Naik, A. 2022^[33]^ | -0.81(-1.18- -0.44) | - | - | Low ^b^ |  |
|  | **SE (95%CI)** |  |  |  |  |
| Tarawneh, AM. 2021^[37]^ | 160.89(32.27-298.50) | 0% | 0.995 | High ^a^ |  |
|  |  |  |  |  |  |
| **LOS** | **MD (95%CI)** |  |  |  |  |
| Fu, WG. 2021^[23]^ | -0.67(-1.16- -0.19) | 67% | 0.01 | Moderate ^a,d^ | Low |
| Li, W. 2020^[29]^ | -0.36(-1.03-0.31) | 62% | 0.07 | Moderate ^a,d^ |  |
| Li, YY. 2023^[30]^ | -1.45(-2.59- -0.31) | 97% | <0.0001 | Very Low ^b,g^ |  |
| Luengo-Matos, S. 2022^[32]^ | -0.36(-1.03-0.31) | 62% | 0.07 | Moderate ^a,d^ |  |
| Siccoli, A. 2019^[35]^ | 0.70(0.20-1.20) | 89% | < 0.0001 | Very Low^b,g^ |  |
|  | **SMD (95%CI)** |  |  |  |  |
| Himstead, AS. 2022^[25]^ | -0.32(-0.42- -0.22) | 79% | <0.01 | Very Low ^b,g^ |  |
| Li, HM. 2020^[27]^ | -0.31(-0.83-0.21) | 70% | 0.07 | Moderate ^a,d^ |  |
| Li, JY. 2020^[28]^ | -0.28(-0.55- -0.01) | 84.4% | <0.0001 | Very Low ^b,g^ |  |
| Naik, A. 2022^[33]^ | 0.57(0.16-0.97) | - | - | Low ^b^ |  |
|  | **SE (95%CI)** |  |  |  |  |
| Tarawneh, AM. 2021^[37]^ | 5.05(4.07-11.17) | 0% | 0.911 | High ^a^ |  |
|  |  |  |  |  |  |
| **IBL** | **MD (95%CI)** |  |  |  |  |
| Fu, WG. 2021^[23]^ | -91.64(-152.44- -30.83) | 89% | <0.0001 | Low ^a,g^ | Very Low |
| Li, W. 2020^[29]^ | -51.47(-112.51-9.57) | 90% | <0.0001 | Very Low ^a,g,h^ |  |
| Li, YY. 2023^[30]^ | -75.64(-140.34- -10.94) | 100% | <0.0001 | Very Low ^b,g^ |  |
| Luengo-Matos, S. 2022^[32]^ | -68.12(-109.24-27.01) | 34% | - | Low ^a,h^ |  |
| Zhou, LP. 2021^[41]^ | -42.49(-78.38- -6.61) | 78% | - | Very Low ^b,g^ |  |
| Naik, A. 2022^[33]^ | 1.83(1.07-2.58) | - | - | Low ^b^ |  |
| Siccoli, A. 2019^[35]^ | 63.0(-13.50-139.50) | 11% | - | Very Low^b,h^ |  |
|  | **SMD (95%CI)** |  |  |  |  |
| Himstead, AS. 2022^[25]^ | -0.25(-0.34- -0.16) | 53% | <0.01 | Very Low ^b,d^ |  |
|  |  |  |  |  |  |
| **VAS** | **MD (95%CI)** |  |  |  |  |
| Fu, WG. 2021^[23]^ | -0.15(-0.54-0.23) | 0% | 0.84 | High ^a^ | Moderate |
| Li, W. 2020^[29]^ | -0.15(-0.34-0.04) | 36% | 0.19 | High ^a^ |  |
| Li, YY. 2023^[30]^ | -0.78(-1.20-0.36) | 99% | <0.0001 | Very Low ^b,d^ |  |
|  | **SMD (95%CI)** |  |  |  |  |
| Li, HM. 2020^[27]^ | -0.17(-0.46-0.12) | 0% | 0.99 | High ^a^ |  |
| Naik, A. 2022^[33]^ | -0.11(-0.67-0.45) | - | - | Low ^b^ |  |
|  | **SE (95%CI)** |  |  |  |  |
| Tarawneh, AM. 2021^[37]^ | 2.85(-2.06-7.78) | 0% | 0.889 | High ^a^ |  |
|  |  |  |  |  |  |
| **ODI** | **MD (95%CI)** |  |  |  |  |
| Fu, WG. 2021^[23]^ | 0.21(-5.09-5.51) | 79% | 0.003 | Low ^a,g^ | Low |
| Li, W. 2020^[29]^ | -2.22(-3.83-0.61) | 3% | 0.38 | High ^a^ |  |
| Li, YY. 2023^[30]^ | -1.49(-2.5-0.48) | 94% | <0.0001 | Very Low ^b,d^ |  |
|  | **SMD (95%CI)** |  |  |  |  |
| Li, HM. 2020^[27]^ | -0.23(-0.52-0.06) | 46% | 0.16 | High ^a^ |  |
| Naik, A. 2022^[33]^ | -0.22(-1.10-0.66) | - | - | Low ^b^ |  |
|  |  |  |  |  |  |
| **Revision rate** | **OR (95%CI)** |  |  |  |  |
| Li, CT. 2021^[26]^ | 0.19(0.05-0.71) | 0% | 0.92 | High ^a^ | Low |
| Li, JY. 2020^[28]^ | 0.38(0.24-0.60) | 0% | 0.934 | Low ^b^ |  |
| Zhou, LP. 2020^[40]^ | 0.25(0.05-1.20) | 0% | - | Low ^b^ |  |
| Zhou, LP. 2021^[41]^ | 0.46(0.15-1.43) | 0% | - | Low ^b^ |  |
| Staartjes, VE. 2018^[36]^ | 0.30(0.10-0.90) | - | - | Low ^b^ |  |
|  | **SE (95%CI)** |  |  |  |  |
| Tarawneh, AM. 2021^[37]^ | 2.46(1.37-3.55) | 90.1% | <0.0001 | Low ^a,g^ |  |

Notes: a, All RCTs Meta-analysis (GRADE assessment begins with High evidence); b, RCTs and None-RCTs Meta-analysis (GRADE assessment begins with Low evidence); c, Risk of bias (going from assessments of risk of bias to judgments about study limitations for main outcomes across all included studies); d, Inconsistency (small P value from a test for heterogeneity or a high I^2^ value, 50%＜I^2^≤75%); e, Indirectness (differences in population, intervention and outcome measures); f, Imprecision (wide 95% confidence interval around the estimate of the effect); g, Severe inconsistency（75%＜I^2^）; h, Severe imprecision (very wide 95% confidence interval around the estimate of the effect); FJV: facet joint violation; IRD: intraoperative radiation dose; IRT: intraoperative radiation time; OT: operative time; LOS: length of stay; IBL: intraoperative blood loss; VAS: visual analogue scale; ODI: Oswestry disability index.
